# Implementing multiple health behaviour change interventions for cardiovascular risk reduction in primary care: a qualitative study

**DOI:** 10.1186/s12875-018-0860-0

**Published:** 2018-10-30

**Authors:** Samah Alageel, Martin C. Gulliford, Lisa McDermott, Alison J. Wright

**Affiliations:** School of Population Health and Environmental Sciences, Faculty of Life Sciences and Medicine, King’s College London, Addison House, Guy’s Campus, London, SE1 1UL UK

**Keywords:** Behaviour change interventions, Primary care, Cardiovascular prevention, NHS health check, Intervention implementation

## Abstract

**Background:**

The implementation of multiple health behaviour change interventions for cardiovascular risk reduction in primary care is suboptimal. This study aimed to identify barriers and facilitators to implementing multiple health behaviour change interventions for cardiovascular disease (CVD) risk reduction in primary care.

**Methods:**

Qualitative study using semi-structured interviews informed by the Theoretical Domains Framework. Interviews were conducted with a purposive sample of healthcare professionals working in the implementation of the NHS Health Check programme in London. Data were analysed using the Framework method.

**Results:**

Thirty participants were recruited including ten general practitioners, ten practice nurses, seven healthcare assistants and three practice managers from 23 practices. Qualitative analysis identified three main themes: healthcare professionals’ conceptualising health behaviour change; delivering multiple health behaviour change interventions in primary care; and delivering the health check programme. Healthcare professionals generally recognised the importance of health behaviour change for CVD risk reduction but were more sceptical about the potential for successful intervention through primary care. Participants identified the difficulty of sustained behaviour change for patients, the lack of evidence for effective interventions and limited access to appropriate resources in primary care as barriers. Discussing changing multiple health behaviours was perceived to be overwhelming for patients and difficult to implement for healthcare professionals with current primary care resources. The health check programme consists of several components that are difficult to fully complete in limited time.

**Conclusions:**

Advancing the prevention agenda will require strategies to support the delivery of behaviour change interventions in primary care. Greater emphasis needs to be given to promoting behaviour change through supportive environmental context. Further research is needed to evaluate current external lifestyle services to improve the intervention outcomes.

**Electronic supplementary material:**

The online version of this article (10.1186/s12875-018-0860-0) contains supplementary material, which is available to authorized users.

## Introduction

Cardiovascular risk management for the prevention of cardiovascular diseases (CVD) through multiple health behaviour change (MHBC) interventions and/or medication has the potential to reduce risk and provide health benefits. The potential for continuity of care in primary care presents an opportunity to support behaviour change interventions. In many countries, behaviour change interventions are being delivered by primary care professionals as part of their routine practice. However, there is only limited evidence for the effectiveness of MHBC interventions at reducing CVD risk and CVD risk factors or improving mortality outcomes among primary prevention populations [1–3]. A Cochrane systematic review evaluated the effect of multiple risk factor interventions on all cause and CVD mortality but did not find an effect on CVD-mortality (Odds Ratio = 0.99, CI = 0.92 to 1.07) [2]. We presently have limited understanding of factors influencing the effectiveness of MHBC interventions in primary care. Understanding providers’ experiences may help to provide explanations for the apparently limited effectiveness of MHBC interventions.

A national health check programme was implemented in England in 2009, targeting individuals aged 40 to 74 years and estimating their risk of developing cardiovascular disease in the next 10 years [4]. After identifying individuals at elevated risk, a risk management intervention, tailored to the individual’s behavioural risk factors, is provided. The risk management component focuses on MHBC intervention to address four behavioural areas: diet and weight management, smoking cessation, promoting physical activity and reducing excessive alcohol consumption [4]. MHBC interventions are defined by the Health Check programme as individual-level behaviour change interventions that can be delivered through brief advice, motivational interviewing and/ or referrals to external behaviour change services [4]. The intervention is delivered in primary care by several different healthcare professionals (HCPs). These include general practitioners (GPs), practice nurses (nurses based in GP surgeries, their main role involves health screening and promotion and managing patients with chronic diseases) and healthcare assistants (HCA) (assistants who help provide care, working under the guidance of a qualified healthcare professional, often a nurse) [5]. The NHS Health Check (NHSHC) programme provides an opportunity in terms of tackling multiple risk factors which, if successful, might reduce the risk of several diseases. However, several limitations were identified in relation to MHBC intervention implementation. A qualitative study examining the experience of high-risk individuals identified gaps in the promotion of behaviour change [6]. High-risk individuals were not always offered behaviour change interventions (especially for physical activity and alcohol consumption) [6]. Additionally, the implementation of behaviour change interventions, when offered, varied across general practices [5, 7]. Failure to deliver personalised behaviour change interventions to high-risk patients in some practices, decreases the likelihood that patients will change their behaviour and reduces any long-term impact of the health check programme [7]. Reported barriers in clinical practice include workload and lack of time, in addition to insufficient knowledge and skills to deliver behaviour change interventions [8, 9]. These barriers may have contributed to the lack of confidence in the effectiveness of behaviour change interventions [8, 9].

Psychological theories have often been used to understand, predict and generate behaviour change in HCPs [10–12]. However, the large number of overlapping psychological theories makes it difficult to select theoretical constructs or models for implementation research. In an attempt to address this problem, Cane et al. (2012) reviewed 128 theoretical constructs derived from 33 theories applicable to implementation behaviour, and grouped these constructs based on commonalities [13]. The Theoretical Domains Framework (TDF) was developed using a consensus approach with groups of experts including health services researchers and health psychologists [13, 14]. The aggregated nature of the TDF provides a programmatic framework for the explanation of HCPs’ behaviours. It has been widely used to investigate perceived barriers and facilitators to HCPs’ evidence-based implementation behaviour [15–17], and shown to be useful for providing explanations for implementation behaviour.

Although existing literature has investigated the experience of healthcare professionals in delivering single behaviour change interventions; further research is needed to understand barriers to the implementation of MHBC interventions. The extent of professionals’ capability and motivation to promote multiple risk-reducing behavioural interventions to high-risk patients, together with the opportunities provided in primary care, influence the success of interventions in reducing CVD risk. This study aimed to explore HCPs experiences of, and views on, delivering MHBC interventions at health checks, in order to identify barriers and facilitators to implementing such interventions effectively in primary care.

## Methods

This study employed qualitative methodology, applying framework analysis to data collected from semi- structured interviews with HCPs who were involved with MHBC interventions as part of the NHSHC programme. The study was approved by King’s College London Research Ethics Committee (LRS- 15/16–2656).

### Recruitment

A purposive sample, aiming to interview participants with a range of professional roles, was recruited from 23 general practices in two socioeconomically deprived and ethnically diverse inner-city London boroughs, Lambeth and Lewisham. The NHSHC uptake in Lewisham (38%) and Lambeth (28%) is below national average (45%) [18]. Of those who received the check, 4% in Lambeth and 8% in Lewisham were classified as at high risk of cardiovascular diseases [19, 20]. These two boroughs have a high proportion of smoking, obesity and excessive alcohol consumption, and high CVD mortality compared to London and England [21]. Most of the health checks conducted are provided in general practices [19, 20].

Potential participants were identified as all HCPs involved in the NHSHC programme implementation. Participants included GPs, practice nurses, HCAs, and practice managers. General practitioners, practice nurses and HCAs carry out the health check assessment, communicate the results and provide MHBC interventions to high risk individuals. Practice managers are involved in managing the business aspect of the practice and support HCPs with delivering patient services. Managers were included in this study as it has been suggested that their interest in and views about health promotion activities act as facilitator to implementation [22]. This heterogeneous sample enabled the identification of common themes across different experiences [23].

Interviews were conducted face-to-face between July and November 2016. Healthcare professionals were contacted via email and invited to take part in the study. Due to clinicians’ busy schedules, the email message was followed by a visit to the general practice two weeks later to encourage participation and answer any enquiries related to the study. When clinicians agreed to participate, a mutually convenient time and place were decided, and consent was obtained before the beginning of each interview. Interviews were conducted by one researcher (SA), with training in qualitative research. Interviews lasted between 25 to 90 min. The length of the interviews varied depending on participants’ experience with the NHSHC and available time. Interviews were audio recoded and transcribed verbatim. Recruitment continued until there were no further emerging themes and it was decided that saturation was reached. Participants were offered a fee for their participation in the study. This study was funded by the National Institute for Health Research (NIHR) Biomedical Research Centre at Guy’s and St Thomas’ NHS Foundation Trust and King’s College London. The funding body had no role in study design, analysing the data or writing the manuscript.

### Interview topic guide

The TDF [13] informed the development of the topic guide (Additional file 1) to identify barriers and enablers to implementing MHBC interventions in primary care. The TDF was developed and widely used for studying the implementation of evidence-based practice by identifying psychological and organizational theory related to clinicians’ behaviour change [13]. The framework provides a robust theoretical basis for studies examining implementation behaviour, covering a broad range of individual and organizational theories [24], so limiting the possibility of missing important factors affecting the implementation of MHBC intervention. Using the TDF to examine barriers and facilitators of HCPs practice might enable the identification of factors that participants may not otherwise report [25].

Developing the topic guide was facilitated by a series of generic interview questions as developed by the originators of the TDF for each domain [13]. At the start of the interview, additional information was collected about participants’ characteristics, such as job title and years of experience of the NHSHC programme. Hypothetical scenarios of high-risk patients were presented to the participants to stimulate the discussion of MHBC interventions. The topic guide was discussed and edited by three experts in the field with experience in using the TDF in qualitative research. It was then piloted with one participant, and the questions were rephrased and rearranged based on the participant’s comments. The transcript from the pilot interview was not included in the analysis, as the aim of the pilot was to improve the topic guide.

### Data analysis

Interview transcripts were managed using NVivo software. Interview data were analysed using Framework analysis [23], as this approach enables data to be compared and contrasted across and within cases until a coherent account emerge [23].

Framework analysis involves seven stages: transcription, familiarisation, coding, developing and applying the analytical framework, charting data into the framework matrix and data interpretation [23] . The familiarisation stage was conducted in parallel to the data collection process. As part of the familiarisation stage, two authors read through the transcripts before coding (SA, LM). The development of the coding framework and initial analysis of interview data was undertaken by the first author (SA) who conducted the interviews. A second author (LM) with expertise in qualitative methods checked all codes to ensure that codes reflected the data. Codes were then discussed, and amendments were made where appropriate until full agreement was reached.

Two authors (SA, AW) assessed and examined the data coded in relation to the TDF. Tables were produced to highlight key thematic content. Factors affecting intervention implementation were assigned to a relevant TDF domain or set of domains [24]. Face to face meetings ensured agreements between authors about charting TDF domains for each factor. Interpretations and over-arching themes were then identified and discussed with the research team.

## Results

Thirty HCPs from 23 general practices in south London took part in the study. The sample included a broad cross section of HCPs, including GPs, practice nurses, HCAs and practice managers. The majority of the sample were female and had been working in delivering the programme since it was introduced. Participants’ characteristics are presented in Table 1. No differences were observed in the experiences of HCPs based on gender, workplace geographical area or length of working in the NHSHC programme.

**Table 1:** Participants characteristics

Gender	
Male	6
Female	24
Job title	
GP	10
Practice nurse	10
Healthcare assistant	7
Manager	3
How long they have been involved with the NHS Health Check	
Since it was introduced in 2009	20
Less than a year	2
More than a year (1–3 years)	8
Borough	
Lewisham	20
Lambeth	10

Three main themes emerged from the data as factors that HCPs viewed as affecting the implementation of MHBC interventions in primary care: 1) Conceptualising health behaviour change; 2) Delivering MHBC interventions in primary care; and 3) Delivering the health check programme. The relationship between themes and the TDF and further illustrative quotes are presented in Table 2.

**Table 2:** Factors influencing the implementation of MHBC interventions for CVD risk reduction

Themes	Sub-themes	Theoretical domain (TDF)	Illustrative quote
Conceptualising health behaviour change	Complexity of health behaviour change	• Beliefs about patients’ capabilities to change their behaviour. • Perceived patients’ environmental context and resources.	“It’s taking us a lifetime to form our behaviours, but they’re expecting us to change overnight. It’s not easy. So we know that changing these behaviours is not easy.” (Interview 15, Manager)
	What is a “healthy behaviour”?	• Patients’ and HCP’s knowledge of health behaviour. • HCP’s emotion towards behaviour change and health behaviour.	“There’s a lot of different conflicting information, even for us in terms of the evidence, it’s still very – so I have to say to my patients, “This is the best I can tell you at the moment, that’s the best information I have.” Will it change? Yes, well it may change. But, you know, we are not saying these are absolute absolutes. This is what we know for the moment.”” (Interview 23, GP)
	Health as a priority	• Perceived patients’ goals to change behaviour. • Perceived patients’ intention to change behaviour.	“So I would kind of usually very much always approach it by kind of where he is at in terms of his attitude to his health and what he feels, you know, he needs to adapt before I even say to him, “Look, here are your figures,” and take him through the meaning or the implications of them. So getting an idea of, of what his understanding is about his health and what concerns he has, to then build on them.” (Interview 20, GP)
Delivering MHBC interventions in primary care	Beliefs about the intervention consequences	• Beliefs about the consequences of implementing behaviour change interventions. • HCPs emotions towards implementing behaviour change interventions. • Reinforcement to implement behaviour change interventions.	“But I think generally the impression I get is the only thing I could tell you is that it’s a waste of time…But yes, generally felt that the brief intervention that you get, what’s the point?” (Interview 29, GP)
	Multiple health behaviour change intervention	• Environmental context and resources that discourages or encourages the implementation of MHBC interventions. • Skills needed to implement MHBC intervention. • Goals.	“I think you can’t obviously deal with everything at once... it would be up to the patient to decide what it is they would like to deal with in the first instance.” (Interview 1, Manager)
	Who should implement health behaviour change interventions?	• Social/professional role and identity. • Social influences. • Skills.	“And I think, I think probably [HCA] because she does more of the health promotion, but she probably has learnt more ways of kind of motivating people and, and has a different relationship with them. So tends to find out a bit about them personally and their family and things. (Interview 24, GP)
	Skills to implement health behaviour change interventions	• HCPs perceived knowledge about MHBC interventions. • Skills needed to implement MHBC interventions. • HCP’s beliefs about their capabilities to implement MHBC interventions.	“In terms of dietary requirements.... yes, it would be nice to just be more specific. Yes. I think, including me, we need more education on dietary advice, for sure.” (Interview 5, HCA)
Delivering the health check programme	The NHSHC programme consists of several steps	• Environmental context and resources. • Skills needed to implement the health check. • Patients perceived knowledge. • Beliefs about capabilities.	“Time is always a major factor. Unfortunately, the GP-land, or practice nursing, as a rule, you’re dealing with everything.” (Interview 6, Nurse) “You can’t give somebody advice if you’re not sure what you’re talking about.” (Interview 14, HCA)
	The health check population	• Behavioural regulation. • Patients’ views of the programme social influences. • Knowledge.	‘But I don’t think that the health check scheme works, because I think it’s targeting the wrong population and it’s, it just - as I said, I think it’s best done opportunistically when we see patients alongside other health issues, which might be more relevant even.’ (Interview 8, GP)

*TDF* Theoretical Domains Framework, *NHSHC* NHS Health Check, *GP* general practitioner, *HCA* healthcare assistant

### Conceptualising health behaviour change

#### Complexity of behaviour change

Participants viewed health behaviour change as a challenging task for their patients. Patients’ ability to change was perceived to be hindered by environmental context and resources (including cost, access to services and time), factors which were not always within patients’ control. The fear of pressuring patients to change their behaviour, when change might not be within patients’ abilities, acted as a barrier to implementing behaviour change interventions.

*“Environmental and societal factors hinder people from changing their behaviour, they’re fighting a losing battle.”* (Interview 21, GP).

Responsibility for enabling behaviour change was discussed. Participants emphasised that regulation at population-level could play a dominant role in facilitating individuals’ behaviour change. Participants expressed the need to make the environment supportive of healthy behaviours, so that subsequently patients will be able to change their behaviour.

*“Unhealthy behaviour is multi-factorial, the reasons why somebody becomes obese are just not as simple as because they eat too much or exercise too little. There are a whole host of socioeconomic and psychological factors. Behaviour change should be facilitated through laws.”* (Interview 8, GP).

#### The nature of a “healthy behaviour”

Participants believed that most patients knew what constituted health-enhancing behaviours and the impact of risk behaviours on their health. Therefore, HCPs perceived little need to explain the importance of changing behaviour to patients.

*“There’s so many messages. I know you can ignore the messages, but people don’t need to be told, “You need to exercise, you need to eat well,” because we all know it.”* (Interview 11, GP).

Some participants were concerned about what healthy lifestyle meant to them and how their own interpretation may be different to that of their patients. Even between HCPs, healthy behaviours were subjective.

*“The definition of healthy behaviour is subjective. people have their own ideas about what good health is.”* (Interview 15, Manager).

#### Health as a priority

For patients to engage in behaviour change, HCPs felt that a healthy lifestyle needed to be on patients’ agendas and a priority in their lives. These goals were seen as a prerequisite to engaging in behaviour change. Healthcare professionals expressed the importance of not imposing their agendas over their patients’ agendas, in order to maintain good relationships with patients and to respond to their wants and needs.

*‘I think that the only way that I’ve experienced for people to actually make changes, is for them to come up with the ideas themselves…using their agenda, not mine.’* (Interview 25, Nurse).

### Delivering MHBC interventions in primary care

#### Beliefs about the consequences of MHBC interventions

Although all HCPs expressed the importance of promoting health behaviour, some were sceptical about the outcomes of MHBC interventions in primary care (see Table 2). The reasons for this scepticism included lack of evidence to support the success of such interventions in primary care and lack of confidence in patients making changes to their behaviour following the intervention.

*“Whether it makes any difference at all to their lifestyle - as we know from the evidence, it doesn’t. In fact, it makes things worse… it’s just a waste of time.”* (Interview 8, GP).

The scepticism was more apparent among GPs, where some compared the success and ease of the “drug approach” in relation to the “non-drug approach”.

*“So I’m very open. I would prefer a non-drug approach to these problems. I think these problems are ultimately based in society and they are behavioural problems… And I know a tablet can’t cure that. And yet the easiest thing for us to do is to prescribe a tablet. And when you do prescribe tablets, tablets do work.”* (Interview 4, GP).

Lack of confidence in the interventions’ success was associated with feeling apathetic and frustrated about implementing MHBC interventions. In contrast, HCPs who were more optimistic about the intervention’s outcomes felt a sense of achievement and satisfaction. Beliefs about the intervention’s consequences were closely linked to HCPs emotions towards intervention implementation.

*“I think we’re slightly apathetic about it from a GP point of view, just because, I don’t know, it’s more soft work that we don’t get a definite outcome from.”* (Interview 11, GP).

*“I think you definitely get a sense, a good feeling of sense of achievement, whatever, if you feel that you’ve really been able to enthuse someone to make some lifestyle changes.”* (Interview 12, Nurse).

Healthcare professionals understanding of MHBC interventions had an impact on how optimistic they were about intervention outcomes. There was a considerable variation in how HCPs viewed the nature of MHBC interventions. Healthcare assistants often referred to behaviour change interventions as involving information sharing; which might explain their positive attitudes towards the intervention. However, GPs felt that patients knew what they needed to change, and therefore needed support to make that change happen, rather than more information. GPs sometimes questioned the long-term impact of behaviour change interventions in terms of maintaining change, which could explain their scepticism regarding the success of the intervention. Therefore, HCPs knowledge and understanding of the intervention and the skills needed to implement it influenced their beliefs about their capabilities and the consequences of implementing MHBC intervention.

*“I’m confident with the information that I give to patients. And they always leave positive.” (*Interview 3, HCA*).*

*“Is it useful? On an individual basis, it may be useful with a particular patient. But on a broader base, is it? I think that’s what often we question. The time to do it, who does it and how well do they do it? In general, I’d say I’m fairly pessimistic because I think it’s difficult.” (*Interview 16, GP*).*

Some participants felt advocating for behaviour change was an essential part of their job, which by itself was an enough of an incentive to implement MHBC interventions (personal incentive). However, monetary (external) incentives were often reported as the main reason to implement the health check among participants who were more sceptical about the intervention’s effectiveness.

*“We’re only doing it, I think, because of the money, because if we don’t, we get the money removed from us and we’ll go under. So I think it is absolutely related to the money. It’s nothing to do with us believing it works or it doesn’t work.”* (Interview 8, GP).

#### Multiple health behaviour change (MHBC) interventions

Addressing MHBC was not always facilitated by primary care resources. Lack of sufficient time to deliver successful behaviour change interventions and to follow-up patients with continuing support were identified as barriers to implementing MHBC interventions.

*“We don’t have loads and loads of time to keep getting people back to give them more advice. But I mean we don’t have a lot of time to do, you know, weight and dietary advice, just dietary advice. There’s a lot of sick people that come in and have to be given a lot of time.”* (Interview 10, Nurse).

Discussing MHBC in one session was often viewed as overwhelming for patients. Participants felt that the best and most successful way to deal with MHBC was for the patient to decide on what to focus and discuss one behaviour change at a time. However, in practice, MHBC change was usually discussed in a single consultation due to contextual constraints (i.e. time and workload).

*“I think in an ideal setting, you would want to tackle, you know, the multiple risk factors, each one of them separately. But there isn’t time to bring patients back for multiple consultations. So you somehow try to discuss all of them.” (Interview 18, GP).*

Healthcare professionals expressed the importance of making referrals to external lifestyle services to support patients through the behaviour change process, but these services had difficulties with long waiting lists, budget cuts causing the discontinuation of some services and were not always offered at times that suited the working population.

*“In terms of what’s available to patients, I think it’s very poor in the UK... And even when they are available, it’s not so accessible, it’s not so easy to negotiate. There’s often a cost involved.”* (Interview 16, GP).

#### Who should implement behaviour change interventions?

Although GPs expressed the importance of behaviour change, they perceived behaviour change interventions as more suitably delivered by nurses and HCAs, who might have more time and appropriate skills, whereas GPs’ time should be reserved for urgent and high-risk cases. Perceived professional role and identity was a barrier to implementing MHBC interventions among GPs.

*“I think then the discussions of lifestyle interventions takes time, which obviously, in today’s work environment, is difficult for GPs. But we do try to train nurses and healthcare assistants to be able to do that as well… I think it’s probably better use of resources to be done by another clinician.”* (Interview 16, GP).

Some GPs were concerned about how promoting behaviour change could affect the balance of their relationship with their patients, which discouraged the initiation of the MHBC discussion. These concerns not clearly held by other HCPs.

*“You have a one-to-one relationship with a patient. It’s too much like the patient has done something wrong, or the patient is weak. It’s interpreted as a weakness if they don’t know how to reduce their diet, if they cannot reduce their smoking…It’s, it becomes less relationship of equals and more relationship of parent-child, good parent, naughty child.”* (Interview 4, GP).

In some interviews, participants discussed how different clinicians influenced the success of behaviour change interventions. Some thought that if the intervention was delivered by a GP it would have a bigger influence on patients. Others argued that patients might be more open and engaged with interventions delivered by nurses and HCAs, due to the ease of the relationship. One manager thought that patients would be more open in community settings rather than with their healthcare provider.

*“But often hearing these healthy lifestyle advices coming from a doctor, often people might value more than from a healthcare assistant.”* (Interview 16, GP).

*“The doctor-patient relationship do affect that because in a community setting, you are in a different setting. The patient tends to be more open in terms of all the other factors that is affecting them, than when they sit in a clinical room.”* (Interview 15, Manager).

#### Skills to implement behaviour change interventions

GPs reported a lack of specific training to implement behaviour change interventions, but some GPs believed that they did not need special training to implement behaviour change interventions. It was viewed as being intrinsic to their medical training.

*“And I think where people like me get really concerned about these questions, “Have you had any training?” Yes, so we’ve been medically trained. I’ve then been trained as a general practitioner. All of the skills required to do a health check are delivered to me. They do not depend on a one-day training programme.”* (Interview 23, GP).

Lack of training was a concern to other HCPs who noted GPs’ competency in managing ill health but not in delivering behaviour change interventions.

*“Well I think clinicians are sort of culturally geared to deal with illness. And dealing with people’s sort of illnesses that they come in with. And less geared up to deal with promoting health and promoting healthy behaviours.”* (Interview 19, Manager).

Some participants described lack of training to deliver specific dietary interventions and individualised behaviour change interventions as barriers to implementation, which may have affected their perceived capabilities to implement the intervention. All HCPs discussed the absence of any guidance on implementing MHBC interventions. It was often perceived as a very individualised approach that should not be structured.

*“You can’t really have a template for the advice. It is kind of depending on each person.”* (Interview 14, HCA).

Most HCPs expressed difficulty in delivering weight management interventions. The fear of offending and upsetting the patient and the difficulty of dealing with such a sensitive subject in limited time discouraged the implementation of weight management interventions. Some HCPs reflected on their own weight and how that affected the way they implemented weight management interventions, either by using themselves as an example to break barriers and facilitate the implementation of behaviour change intervention or that their weight might have inhibited the process.

*“And they relate to me. You know, clearly I’m not thin. So when they sit down and I say, “You weigh a little bit more than is good for you,” I say, “And you weigh a little bit more for you than is good for you, like I do.” You know, “You’re not the only one that has those problems.””* (Interview 25, Nurse).

Excessive alcohol consumption was also considered a difficult behaviour to tackle. Some HCPs thought patients would not easily discuss their alcohol consumption and its part in their social life. Issues around assessing patients’ alcohol consumption accurately were also reported as barriers to implementing alcohol consumption interventions.

*“The most difficult one I find is talking about the alcohol, because kind of some people aren’t kind of upfront about the amount of alcohol.... So I find that the hardest really.”* (Interview 9, HCA).

### Delivering the health check programme

#### The programme consists of several steps

The majority of HCPs expressed difficulty in going through all the health check steps within one consultation.

*“One of the problems has been, as with any service, by putting too many steps in, you can lose, you know, people get lost. So we want to try and tighten that up…One of the challenges of the health check, I think, is it’s got complicated assessment. I think it’s quite a big ask.”* (Interview 1, Manager).

A further challenge was in discussing the results of the checks’ several components, including CVD-risk score, in limited time. Comprehending CVD risk was perceived to be challenging for patients, where HCPs have expressed concerns over the inconsistency between patients’ perceived risk and their actual risk.

*“I think it’s virtually an impossible explanation to give.” To say somebody, “You have a risk in the next decade of dying,” it’s impossible. It’s just impossible to say to somebody, “Your QRISK is...this is based on a population sample.”* (Interview 8, GP).

#### The health check population

Patients who responded to the health check invitation were perceived to be healthy and at low risk. Healthcare professionals rarely identified high-risk patients through the health check invitation process, and patients checked opportunistically were perceived as the target group.

*“I sometimes think we only get the ones booking, who want to do something - the ‘worried well’ I call them. Yes, whereas, I think the ones we pick up opportunistically are the more needy. And actually they’re far harder to work with.”* (Interview 12, Nurse).

Participants expressed concern over patients’ perceptions of the programme (Table 2). Most patients who came for their check were thought to not understand the programme rationale. Patients’ lack of knowledge about the programme’s intended outcome of reducing risk was perceived as a barrier to patients’ willingness to change their behaviour.

*“And they often come in not quite sure what it’s all about, but knowing that it’s a health check… But yes, so patients will see it as a, ‘just a check to see that I’m healthy, to see that I’m okay.’”* (Interview 10, Nurse).

## Discussion

This study is among the first to examine HCPs’ experiences of the implementation of MHBC interventions as part of cardiovascular health checks in primary care. The study identified several key domains that either facilitate or discourage the implementation of behaviour change interventions.

Healthcare professionals were concerned that behaviour change interventions delivered in primary care might be over-reliant on patients’ individual agency, given many environmental factors can hinder efforts at behaviour change. They discussed the broader socioeconomic context in which behaviour change occurs and the need for supportive policies and regulation. The lack of a supportive wider context led to HCPs doubting the effectiveness of behaviour change interventions delivered in primary care and being less motivated to implement them. They also felt that such interventions may be an unfair imposition of HCPs’ agendas on patients who were unable to change, given patients’ circumstances. The importance accorded by HCPs to societal influences on behaviour contrasts with views of GPs in the same area of London over a decade ago, who saw obesity-related behaviour change as being chiefly the patient’s responsibility [26]. There has been recent criticism of the public health interventions favoured by policymakers as over-reliant on individuals’ ability to draw on personal resources to change. Critics have argued that interventions requiring a lower level of agency to benefit will be more effective and equitable [27]. Healthcare professionals may be more likely to be willing to promote behaviour change at an individual level when interventions in primary care are accompanied by efforts to make the wider environment more facilitative of health enhancing behaviour.

Healthcare professionals in our study questioned the effectiveness of behaviour change interventions delivered in primary care, especially in the long-term. These perceived lack of effectiveness of behaviour change was contrasted with the “drug approach” to risk reduction, which was seen as easier and more effective. However, when HCPs felt they had personal experience of successfully supporting patients to change behaviour, their sense of achievement motivated further efforts to deliver behaviour change interventions. In contrast, perceiving interventions as ineffective led to frustration and apathy. Although a recent systematic review did not find that MHBC interventions in primary care had quantitatively important effects on CVD risk factors [1], the included interventions often did not incorporate behaviour change techniques [28] now known to promote behaviour change. Sharing evidence about specific, effective behaviour change techniques, may increase HCPs’ willingness to deliver behaviour change interventions that incorporate them. Several behaviour change techniques have been shown to be effective in changing behaviour in primary prevention populations and could be easily implemented in primary care setting. For example, prompting a plan to perform the behaviour (i.e. action planning) have been shown to increase physical activity [29, 30] and advice on stop smoking medications have been shown to facilitate smoking cessation [31]. The provision of smoking cessation medication is considered an effective strategy in sustaining smoking cessation, even among smokers who are not ready to quit smoking [32, 33]. In the present study, perceiving low effectiveness did not appear to deter HCPs completely from delivering health checks, due to the financial incentives for conducting the checks making an important contribution to GP practices’ budgets. However, it may be harder for HCPs who feel doubtful about the effectiveness of behaviour change interventions to enthuse patients to change their behaviour.

Environmental context and resources were suggested to discourage the implementation and follow-up of MHBC interventions in primary care. Time constraints and workload pressures in general practice were viewed as barriers to implementation, as reported previously [22, 34]. In the NHSHC programme, clinicians are expected to assess people’s risk, communicate the results and provide MHBC intervention in one session lasting at most 30 min. In contrast, HCPs felt that tackling one behaviour at a time, and being able to see patients for follow-up sessions, was more likely to promote behaviour change. Therefore, there is a need to develop and disseminate very brief behaviour change interventions that could be incorporated into health checks. For example, Pears and colleagues evaluated three very brief interventions to promote physical activity. The results suggested that providing a pedometer with instructions on how to use it and explanation of the recommended steps per day would take only five minutes and result in increased physical activity levels [35]. Technological solutions could enable patient follow-up without requiring further appointments. Many GP practices in England already use a text messaging system to contact patients with appointment reminders and cues for preventive health care. This system could also be used to further deliver brief behaviour change intervention components or follow-up on whether patients have met behavioural goals and provide suitable feedback. Providing HCPs with guidance on using technology-based methods of follow-up may facilitate intervention implementation. The usefulness of the guidance, however, is limited by professionals’ beliefs of the importance of the implementation and effectiveness of such interventions [22].

Given the limited time available in primary care, referrals to community behaviour change services and resources may be provide a more suitable platform for behaviour change [36]. However, HCPs noted a number of difficulties with referring patients to these services. Some services had long waiting lists, or were even no longer available due to budget cuts, while others did not run at times convenient for a working age population. There is therefore a need to ensure these services are properly resourced and made available at times that reflect the availability of the working age health check population. However, as it would be difficult to offer community services at times to suit all eligible individuals, digital behaviour change interventions may provide an alternative, easily scalable and convenient solution. Therefore, research attention should also be paid to establishing the most effective digital interventions for the health check population.

Feeling one lacked the necessary skills and training made HCPs doubt their effectiveness at promoting particular types of behaviour change. Weight management interventions were inhibited by fears of upsetting patients, while alcohol reduction interventions were seen as difficult because patients might be uncomfortable disclosing the true extent of their drinking. Therefore, there is a need for further training and resources to enable clinicians to be more confident in tackling these two sensitive topics. In contrast, some HCPs felt that behaviour change was easy to promote. However, these HCPs seemed to view behaviour change as largely involving information provision. Therefore, there is a need to disseminate resources highlighting behaviour change techniques that are more effective than information provision but still brief.

### Strengths and limitations

This study is among the first to present qualitative findings relating to HCP’s experience in implementing MHBC interventions after health checks in primary care. It examined the views of a diverse group of clinicians and managers who work in the NHSHC programme. Using the TDF may have led participants to discuss the role of less conscious factors, such as emotions, that may influence their implementation behaviour [25]. The TDF synthesizes 33 theories of behaviour [13], so it is likely to be comprehensive in comparison to studies based on a single theory or using a conventional topic guide. The TDF enables looking beyond individual factors and considers environmental and social influences on implementation behaviour. The interviews were coded and analysed by two authors with experience in qualitative research and background in health psychology, which improves the reliability of this study interpretation [37].

The findings of this study may not be completely transferable beyond the current context. The results reflect the views of those who agreed to participate and who work in specific geographical area. Healthcare professionals who were not able to participate may have different experiences. Face to face interviews can produce social desirability bias [38], especially when discussing implementation behaviour. Ethnography could have provided the advantage of considering what HCPs do and whether it is similar (or not) to what they say they do. The interviewer was external to the practice and other related agencies, with no conflicting roles or affiliations, which is believed to help in accessing more private accounts and reducing socially desirable responses. Finally, it was difficult to reach theoretical saturation of themes representing the manager group, considering the limited number of managers in the two boroughs. However, it is believed that including this group provided further insights on possible factors that affect the implementation of MHBC interventions.

## Conclusion

This study has identified several factors that may restrict the effective implementation of MHBC interventions for cardiovascular risk reduction. The implementation of MHBC interventions should be facilitated by the provision of suitable training in behaviour change techniques and specific approaches to very brief behaviour change interventions. Healthcare professionals should be supported with easy, efficient and effective guidance to implement MHBC interventions given limited resources. The use of technology-based interventions to facilitate behaviour change could also be considered. Greater emphasis on promoting behaviour change through supportive environments and external lifestyle services may improve behaviour change outcomes.

## Additional file

Additional file 1: Topic guide. Includes interview questions. (DOCX 28 kb)

## Abbreviations

CVD: Cardiovascular disease
GP: General practitioner
HCA: Healthcare assistant
HCP: Healthcare professional
MHBC: Multiple health behaviour change
NHS: National Health Services.
NHSHC: National Health Services Health Check
TDF: Theoretical Domains Framework
