# Supplementary material for: Implementing multiple health behaviour change interventions for cardiovascular risk reduction in primary care: a qualitative study
Source: BMC Fam Pract. 2018 Oct 30;19:171. doi: 10.1186/s12875-018-0860-0 (PMC6208114; doi:10.1186/s12875-018-0860-0)
Supplement: Supplementary file 1 — Topic guide. Includes interview questions. (DOCX 28 kb) [file 12875_2018_860_MOESM1_ESM.docx]

**Topic Guide**

**At the beginning of the interview:**

- Thanking the participant for his/her time, and for participating in this research.
- Introducing myself.
- Asking participant to re-read the information sheet, address any questions and ask them to sign the consent form.
- Confirm the permission to record.

**General questions regarding the participants:**

- Job title.
- How long have you been working in the NHS Health Checks programme?
- Their role in the programme.
- Any special training related to the program itself.

**I would like to start with general questions about the NHS Health Checks programme and then we would focus on the lifestyle advice (i.e. risk management) part of the programme.**

**Environmental context and resources:**

Can you tell me about the set-up for health checks in your practice?

- Probe: the health check process?

What are the responsibilities of different staff members in relation to implementing the programme?

- Probe: do you feel it’s clear who should do what?

**Knowledge:**

What is your understanding of the lifestyle advice part of the NHS Health Check?

Are there any protocols or guidance to facilitate lifestyle advice?

- Probe: for the programme in general, within the practice.

If present, what are your thoughts about the guidance?

- Probe: do you agree with it? Does it provide enough details?

In your opinion, what do you think are the best ways to influence behaviour change in people who’ve had a health check?

- Probe: Are there particular approaches that you think are or might be useful?

**Skills (clinical scenarios):**

If you were presented with this case (case scenario #1 or #2 below), what would you do?

- Probe: what lifestyle advice you would give? How?
- Probe: how would you follow-up with this patient? how would you evaluate the lifestyle advice?

What skills do you think are required in order to provide effective lifestyle advice?

**Intention:**

Is lifestyle advice something you generally intend to do during NHS Health Check consultations?

**Beliefs about capabilities:**

How difficult or easy is it for you personally to provide lifestyle advice?

What makes you say that?

What would help you overcome these difficulties?

- Probe: training? if so, in what?
- Probe: time? If so, how much more?
- Probe: reminders/prompts?

**Professional role and identity:**

Do you think it is an appropriate part of your job to provide lifestyle advice?

**Beliefs about capabilities:**

Are the necessary resources available to the people expected to give patients lifestyle advice?

Are there competing tasks or time constraints that affect whether or how patients are given lifestyle advice?

**Optimism:**

How confident are you that giving lifestyle advice to people who have had a health check, will generally work out well?

Probe: What makes you say that?

**Memory, attention and decision processes:**

Is providing lifestyle advice something you would routinely do as part of the health check, or do you make a deliberate decision about whether to do so for each patient?

- Probe: What factors affects your decision to provide lifestyle advice?

**Goals:**

When patients have several lifestyle risk factors, do you tend to try to tackle them all at once or start with just one or two?

- Why is that? Do you think it’s more helpful….
- Probe [if start with one/two behaviours only] what do you consider when deciding which lifestyle risk factors to tackle first?

Do you set goals for yourself or your practice with regards to managing the lifestyle risk factors of health check patients?

**Reinforcement:**

Are there any incentives that affect whether or how you provide lifestyle advice to health check patients?

**Social influences:**

What do other practice team members think of the NHS Health Check? And about providing lifestyle advice as part of the health check?

Do these views influence whether you offer lifestyle advice or not?

How about the patients? How do you think they feel about having a NHS Health Check?

Probe: Do you think that patients expect you to offer lifestyle advice as part of the health check?

Do these views influence whether you offer lifestyle advice or not?

**Behavioural regulations:**

Is implementation of the lifestyle advice portion of the health check monitored in any way within your practice?

Are there procedures or ways of working in your general practice that encourage offering lifestyle advice to people undergoing NHS health checks?

**Beliefs about consequences:**

What do you think will happen if you provide health check patients with lifestyle advice?

- Probe: how do you think patients will react?
- Probe: do you think patients will alter their lifestyles?

What do you think you will happen if you refer patients to other services that support lifestyle change?

- Probe: Will patients take up the referrals? Why or why not?
- Probe: smoking cessation services?
- Probe: Lifestyle hub (if interviewing practices eligible to refer their patients to the hub)

**Emotion:**

Does providing patients with lifestyle advice evoke an emotional response in you?

- Probe: in what way?

**At the end of the interview:**

That all the questions I had for you today. Has anything else occurred to you about this topic that we haven’t asked about?

Overall, what were your thoughts about the interview?

Thanking the participant for his\her time and participation.

**Case scenario #1:**

You have an appointment with Mr. Johnston, here is the information you have based on the health check.

| Gender | Male |
| --- | --- |
| Age | 60 |
| Marital status | Married |
| Ethnicity | White |
| Family history of coronary heart disease | No |
| Cholesterol ratio | 6.5 mmol/L |
| Systolic blood pressure | 165 mmHg |
| Height | 170 cm |
| Weight | 90 kg |
| BMI | 31 kg/m^2^ |
| Smoking status | Moderate smoker (10-19 cigarettes) |
| Diagnosed with diabetes | No |
| QRISK2 | 26.1% |

**Case scenario #2:**

You have an appointment with Mr. Hussain, here is the information you have based on the health check.

| Gender | Male |
| --- | --- |
| Age | 47 |
| Marital status | Married |
| Ethnicity | Pakistani |
| Family history of coronary heart disease | No |
| Cholesterol ratio | 5.9 mmol/L |
| Systolic blood pressure | 150 mmHg |
| Height | 175 cm |
| Weight | 80 kg |
| BMI | 26 kg/m^2^ |
| Smoking status | Ex-smoker |
| Diagnosed with diabetes | No |
| QRISK2 | 11.1% |
